# Superconductivity of the Ga-Ag Eutectic Alloy Under Nanoconfinement

**DOI:** 10.3390/nano16211444

**Published:** 2026-11-08

**Authors:** Marina V. Likholetova, Elena V. Charnaya, Igor A. Kasatkin, Yurii A. Kumzerov, Alexander V. Fokin

**Affiliations:** 1 St. Petersburg State University, Research Park, 199034 St. Petersburg, Russia; igor.kasatkin@spbu.ru; 2 Physics Faculty, St. Petersburg State University, 199034 St. Petersburg, Russia; e.charnaya@spbu.ru; 3 Ioffe Physical-Technical Institute, Russian Academy of Sciences, 194021 St. Petersburg, Russiamidbarzin@yandex.ru (A.V.F.)

**Keywords:** superconductivity, gallium alloys, Ga-Ag, nanocomposites, ferromagnetism

## Abstract

Gallium alloys are a major focus of modern research due to their applications in emerging fields such as microelectronics, soft robotics, wearable devices, and biotechnology. We present an investigation into the effects of nanoconfinement on superconductivity in a Ga-Ag alloy with a composition close to the eutectic point on the gallium-rich side. Magnetization measurements were carried out within a temperature range of 1.8 to 10 K for two nanocomposites based on synthetic opal and porous glass matrices loaded with Ga-Ag. Superconducting transitions were observed at temperatures significantly higher than the critical temperature of bulk gallium, allowing us to construct the corresponding superconducting phase diagrams. Furthermore, superconductivity was found to coexist with ferromagnetism, the appearance of which is ascribed to the interface between gallium and Ag_3_Ga_2_ segregates. The ferromagnetism induces a shift between the branches of the magnetization isotherms. Finally, a thermal hysteresis was observed between the temperature-dependent magnetization curves measured under field-cooled cooling (FCC) and field-cooled warming (FCW) protocols.

## 1. Introduction

Gallium-based materials have attracted considerable attention owing to their potential in rapidly emerging areas of flexible microelectronics, soft robotics, wearable devices, and biotechnology [1,2,3]. The recent trend toward miniaturization is reflected in [4,5,6,7]. At the nanoscale, gallium and eutectic gallium alloys have been shown to form different crystalline polymorphs [8,9], which show lower melting points, remarkable supercooling, and enhanced superconducting critical temperatures. Polymorph transitions (liquid–liquid phase transitions) were also observed in liquid Ga, Ga-In, and Ga-In-Sn under nanoconfinement [10,11]. At low temperatures, superconducting gallium and its alloys serve as self-healing low-loss circuits [12,13]. They sustain stronger magnetic fields allowing for the construction of compact field meters and sensitive particle detectors. Gallium nanoparticles were suggested to be spread evenly over semiconductor host layers to form a network of Josephson junctions [14,15]. Current pulses trigger transitions in the network, yielding low-power cryogenic switches. Nanoparticles of eutectic gallium alloys suspended in solvents create printable superconducting inks for cryo-electronics [16].

Gallium and gallium alloys embedded into nanoporous rigid silica or polymer templates form nanocomposites with a tuned morphology of the metallic network. The dendritic shape and connectivity of confined nanoparticles are mainly driven by pore geometry [17]. Superconductivity is strongly affected by nanoconfinement. Rigid silica nanoporous matrices filled with gallium showed type-II behavior, whereas bulk gallium is a type-I superconductor, as are most pure metals [18]. The change in the type of superconductivity is induced by a reduction in the correlation length due to the scattering of conduction electrons by nanoparticle boundaries. Features of nanoconfined gallium can differ from those of relevant isolated nanoparticles as they are additionally influenced by coupling with pore walls and links between the metal in neighboring pores. The large total interface between the confined gallium surface and the pore volume was generally considered a crucial factor leading to the specific behavior of nanocomposites based on porous templates. For eutectic gallium alloys, the role of this factor should increase due to the interface between segregates in solid alloys. The shape of the nanoconfined segregates is governed by pore size and alloy composition. These properties were studied for the gallium–indium alloys by small angle neutron scattering and computer simulation [19].

The extended interface between segregates in a specific nanoconfined alloy may induce unusual properties, which cannot be expected in bulk. A unique ferromagnetism was found in the In-Ag eutectic alloy embedded into a porous glass [20,21]. This ferromagnetism coexisted with superconductivity at low temperatures, yielding shifts of the branches of the magnetization isotherms below the superconducting transition. The emergence of ferromagnetism was ascribed to the interface between indium and intermetallic AgIn_2_ segregates. Note, that ferromagnetism has recently been observed in thiol-capped silver nanoparticles (see [22] and references therein).

Magnetic studies of superconductivity were reported in [23,24] for the Ga-Ag alloy embedded into two different porous glasses with pore sizes of 10 and 7 nm. The alloy composition, 3 at.% Ag, was close to the eutectic point in the bulk alloy. Drastically different results were obtained in these two studies. A single superconducting transition was observed in [24] with a critical temperature of 7.03 K at 10 Oe. The field screening at low magnetic fields was near complete, showing that almost the entire nanocomposite was enveloped by superconducting currents. The high temperature of the superconducting transition was treated under the assumption of polymorphism induced by nanoconfinement. In contrast, three superconducting transitions at 7.05, 6.08, and 2.65 K at 10 Oe were found in [23]. The screened volume of the sample was small below the first two transitions and increased remarkably below the third one. While the transitions near 7 and 6 K were likely related to gallium polymorphs, the transition at the lower temperature was ascribed to a gallium–silver intermetallic. Unusual thermal hystereses of magnetization were observed for both nanocomposites, revealing various behaviors of the superconducting vortices.

In the present work, we used dc magnetometry to study superconductivity in two nanocomposites consisting of synthetic opal and porous glass templates loaded with the Ga-Ag alloy. The alloy composition was close to the eutectic point in the bulk alloy. The templates differ from the porous glasses employed in [23,24]. This allows us to expect more information on the impact of pore-network geometry on gallium polymorphism and superconductivity under nanoconfinement. Transitions associated with successive onsets of diamagnetism were observed in the nanocomposites, with distinct critical temperatures for the two samples. Phase diagrams revealed that one of the critical lines for both nanocomposites demonstrated segments with positive and negative curvatures. Ferromagnetism was found in both nanocomposites up to room temperature, which was not reported in [23,24]. The ferromagnetism coexisted with superconductivity at low temperatures, producing shifts of the branches of the magnetization isotherms, which have so far been reported only for a nanocomposite with the eutectic In-Ag alloy [20,21]. The thermal hystereses due to pinning vortices were seen between the magnetization branches obtained upon cooling and warming in the magnetic field.

## 2. Materials and Methods

**Figure 1:**
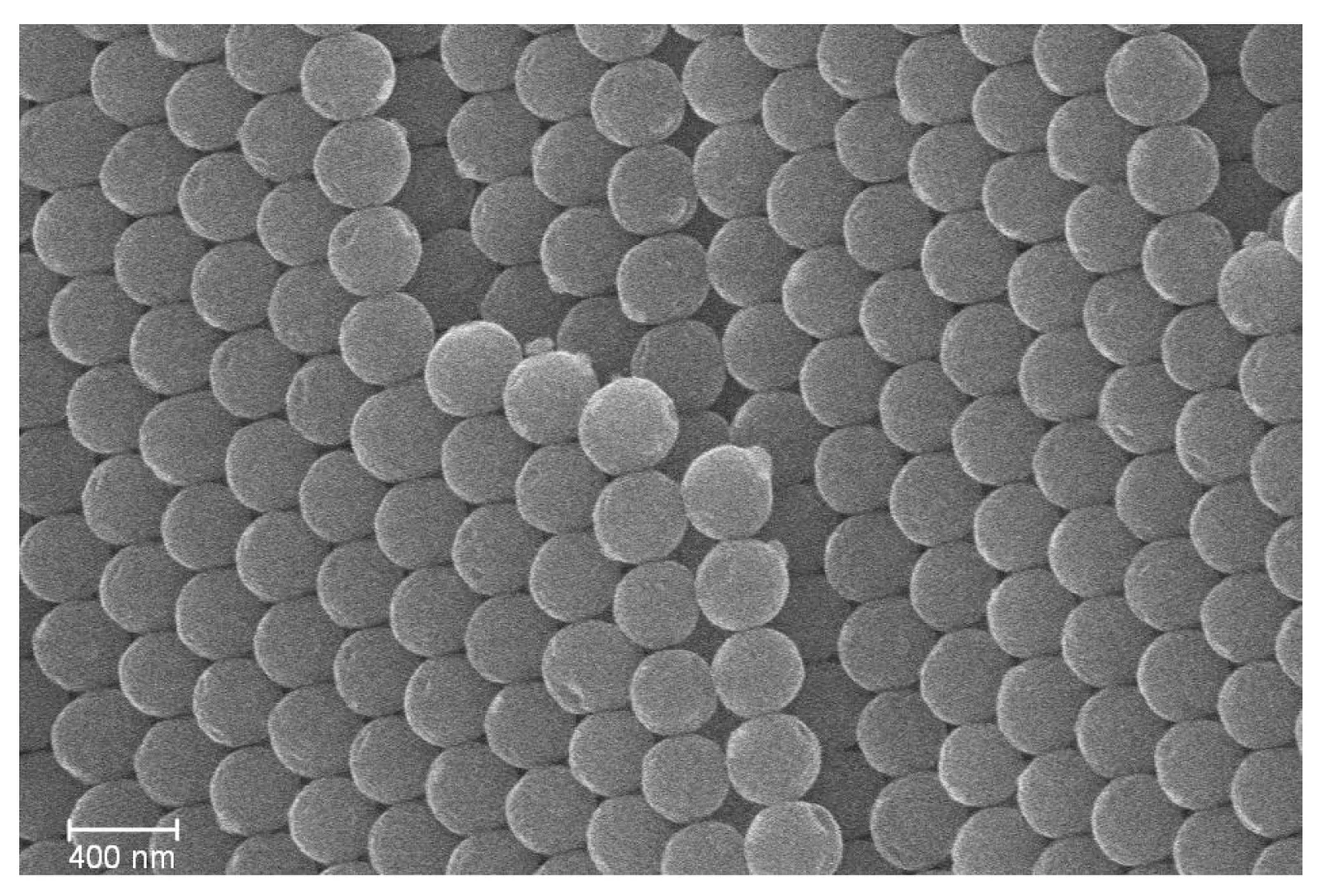
SEM image of the opal matrix.

The synthetic opals have a cubic close-packed structure of amorphous silica spheres. The sphere diameter of 350 nm was determined using a Zeiss Merlin scanning electron microscope (Carl Zeiss Microscopy GmbH, Jena, Germany) (Figure 1). There are two kinds of pores between the cubic close-packed spheres: octahedral and tetrahedral ones. The pore sizes in the ideal opal structure are approximately 145 and 79 nm, respectively. The total pore volume is near 26%. The pore sizes can be reduced by sintering the opal matrices.

**Figure 2:**
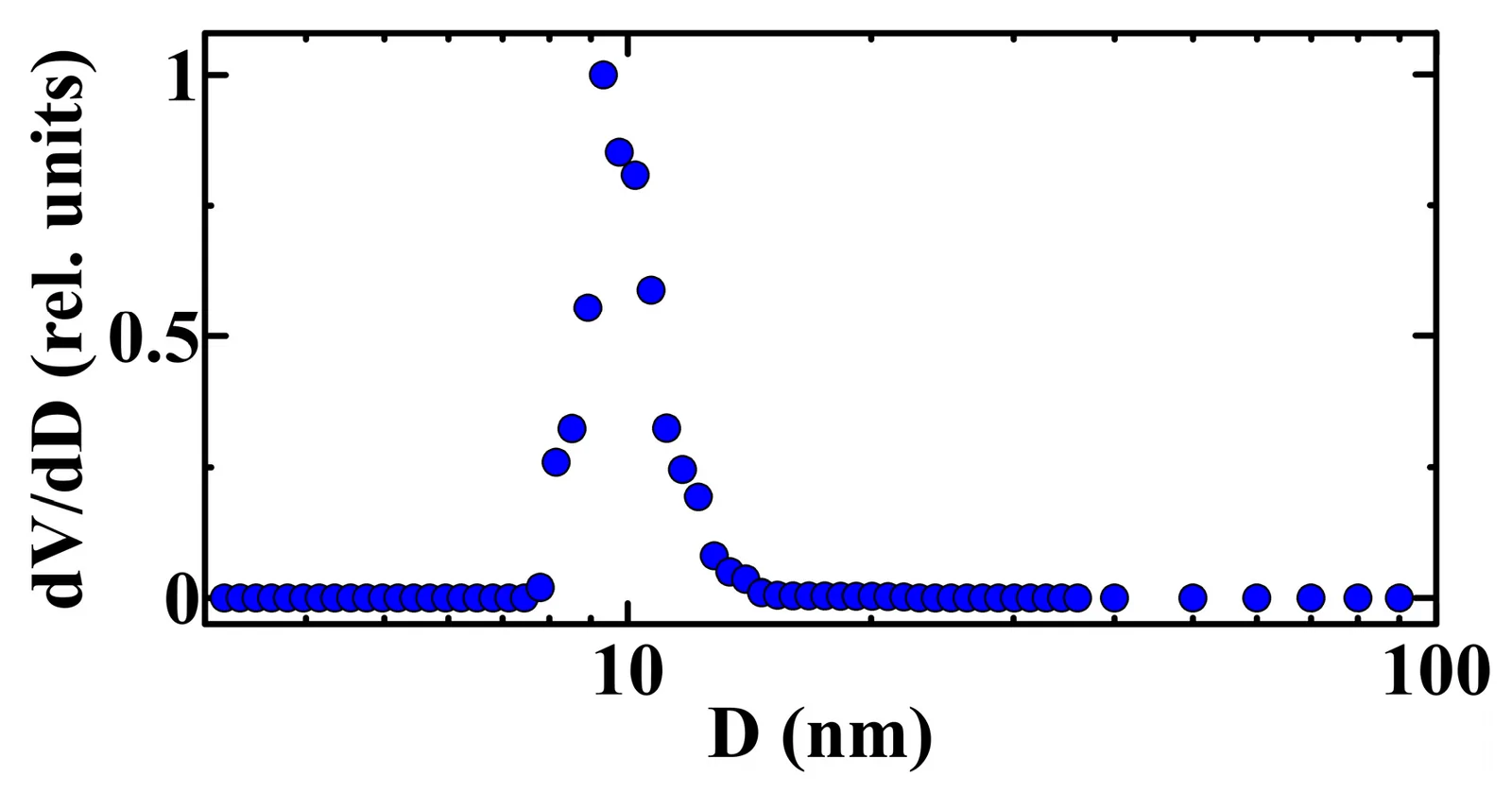
Pore size distribution for the porous glass matrix.

The porous glass template was a boron–sodium silica glass whose pore network was made by phase separation and acid leaching. The mean pore size of 9 nm and the total pore fraction of 23% were obtained by nitrogen porosimetry using a Quadrasorb SI analyzer (Quantachrome Instruments, Boynton Beach, FL, USA) (Figure 2).

The binary gallium–silver alloy consisted of 97 at.% Ga and 3 at.% Ag. This composition is close to the eutectic point in the bulk alloy in the gallium-rich corner [25,26]. The purity of the alloy components was 99.99%. The molten Ga-Ag alloy was embedded into the pores of the opal and glass templates under pressure of up to 20 kbar. The pore occupancy of about 90% was estimated by weighing the porous templates before and after packing. The thin plates for measurements were cut from the filled templates. Their surfaces were cleaned to prevent any influence of the bulk alloy. The weights of the filled opal and porous glass plates were 9.89 and 14.30 mg, respectively.

The dependences of the dc magnetization *M* on temperature and the *M*(*H*) isotherms were measured using a MPMS 3 SQUID-VSM magnetometer (Quantum Design, San Diego, CA, USA). The main measurements were carried out within a temperature range from 1.8 to 10 K at applied fields up to 70 kOe. The plates were oriented parallel to the magnetic field. Three protocols were used to obtain the temperature dependences of magnetization at various magnetic fields. First, the samples were cooled to the lowest temperature limit in zero field. Then, the field was applied and measurements were performed on warming (ZFC), on cooling (FCC), and on subsequent warming (FCW). The magnetization isotherms were obtained by increasing the field from zero to 70 kOe, decreasing it to −70 kOe, and then increasing it again to 70 kOe.

**Figure 3:**
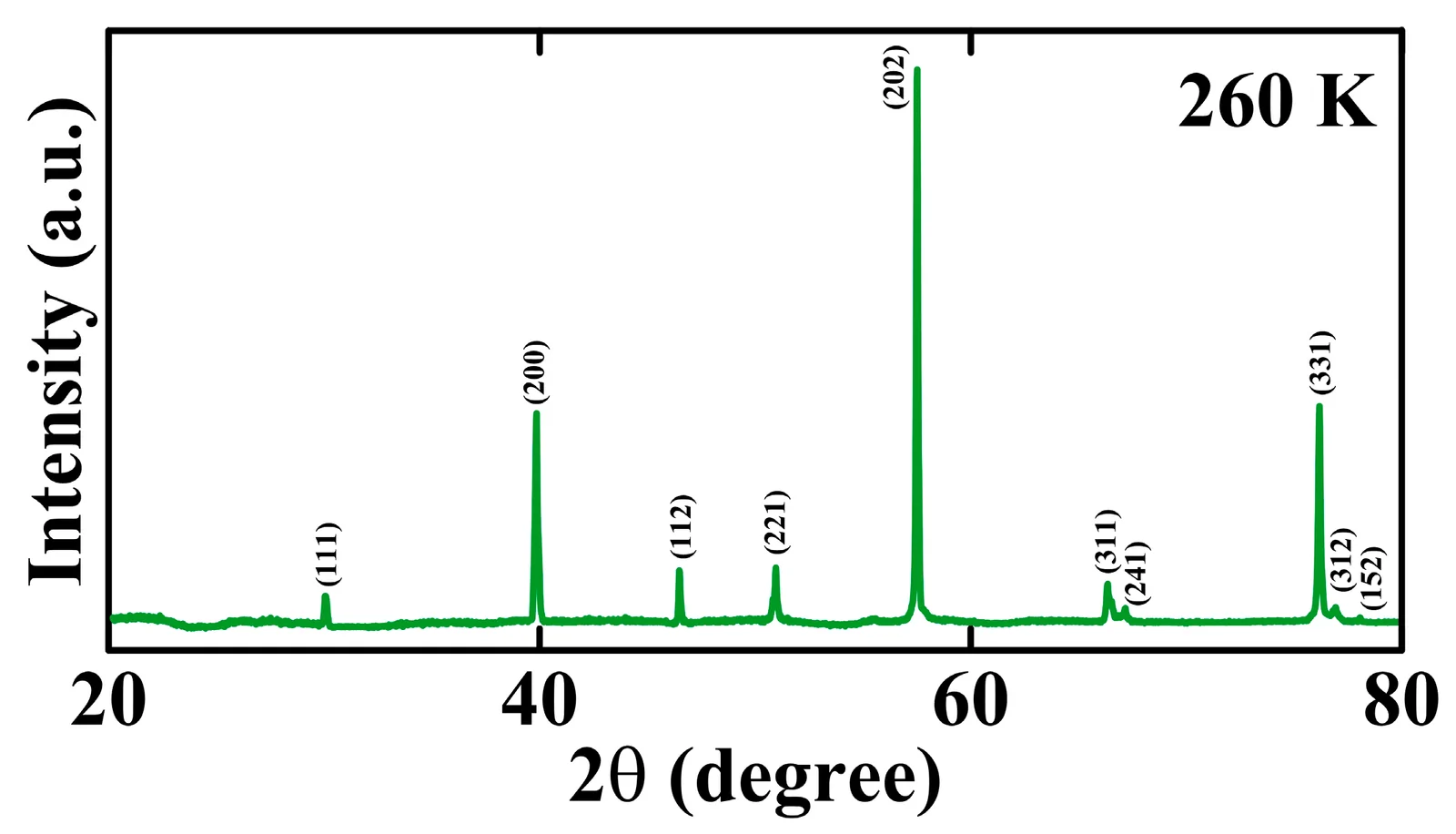
X-ray powder diffraction pattern for the opal nanocomposite.

X-ray powder diffraction studies were conducted using an Ultima IV–185 CuK_α_ diffractometer (Rigaku Corporation, Tokyo, Japan) on cooling within the temperature range from 297 to 110 K. Figure 3 presents the X-ray pattern for the opal nanocomposite at 260 K. The peaks are associated with α-Ga (orthorhombic symmetry, space group *Cmce*). No other peaks were observed on cooling down to 110 K within experimental accuracy.

**Figure 4:**
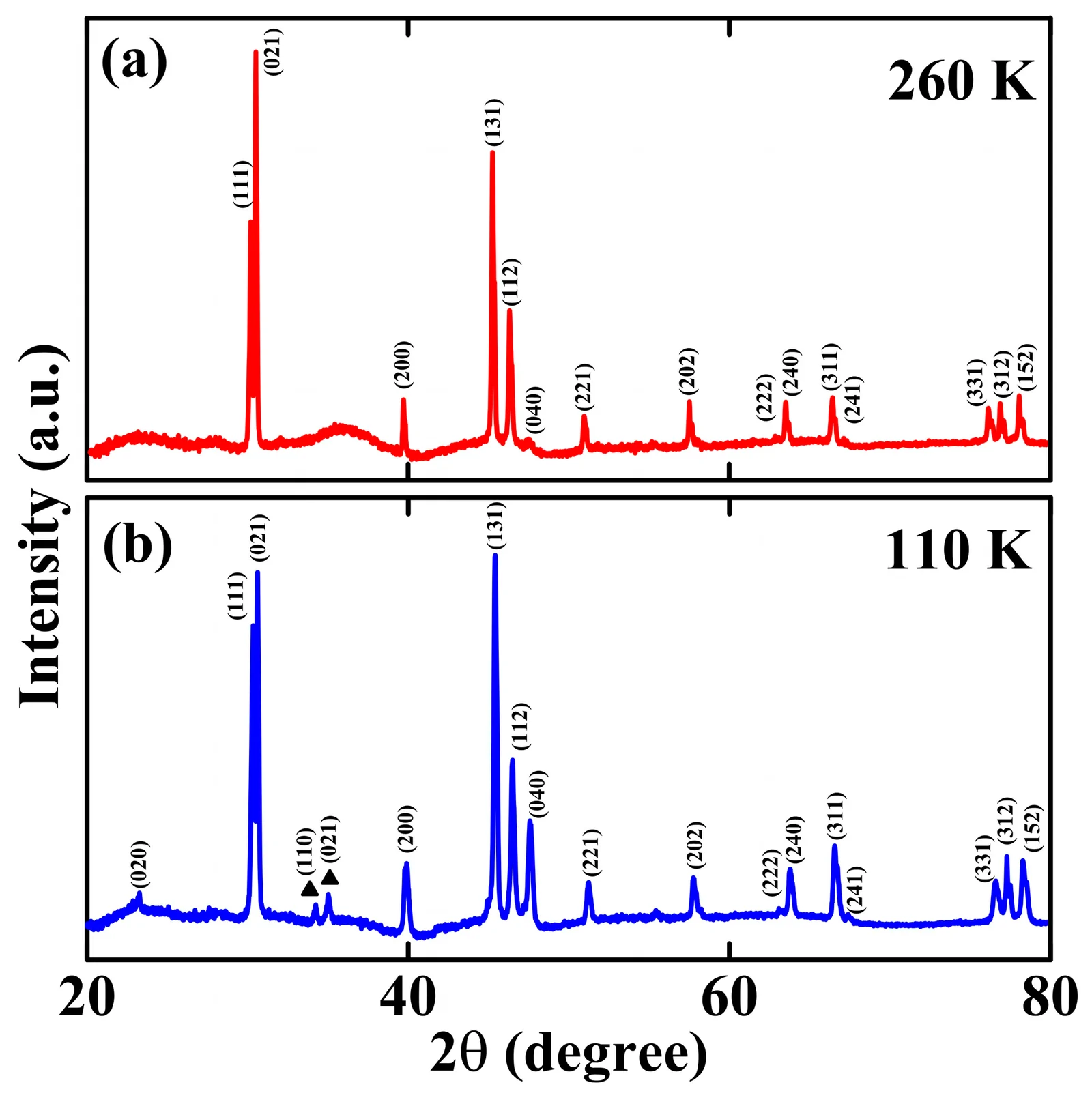
X-ray powder diffraction patterns for the porous glass nanocomposite at: (**a**) 260 K; (**b**) 110 K. Triangles indicate the β-Ga peaks.

Figure 4 shows the X-ray patterns for the porous glass/alloy nanocomposite at 260 and 110 K. The pattern at 260 K comprises the α-Ga peaks (Figure 4a). Two additional peaks emerged below 180 K, which are associated with β-Ga (monoclinic symmetry, space group *C*2/*c*). The pattern remained the same down to 110 K (Figure 4b).

## 3. Results and Discussion

**Figure 5:**
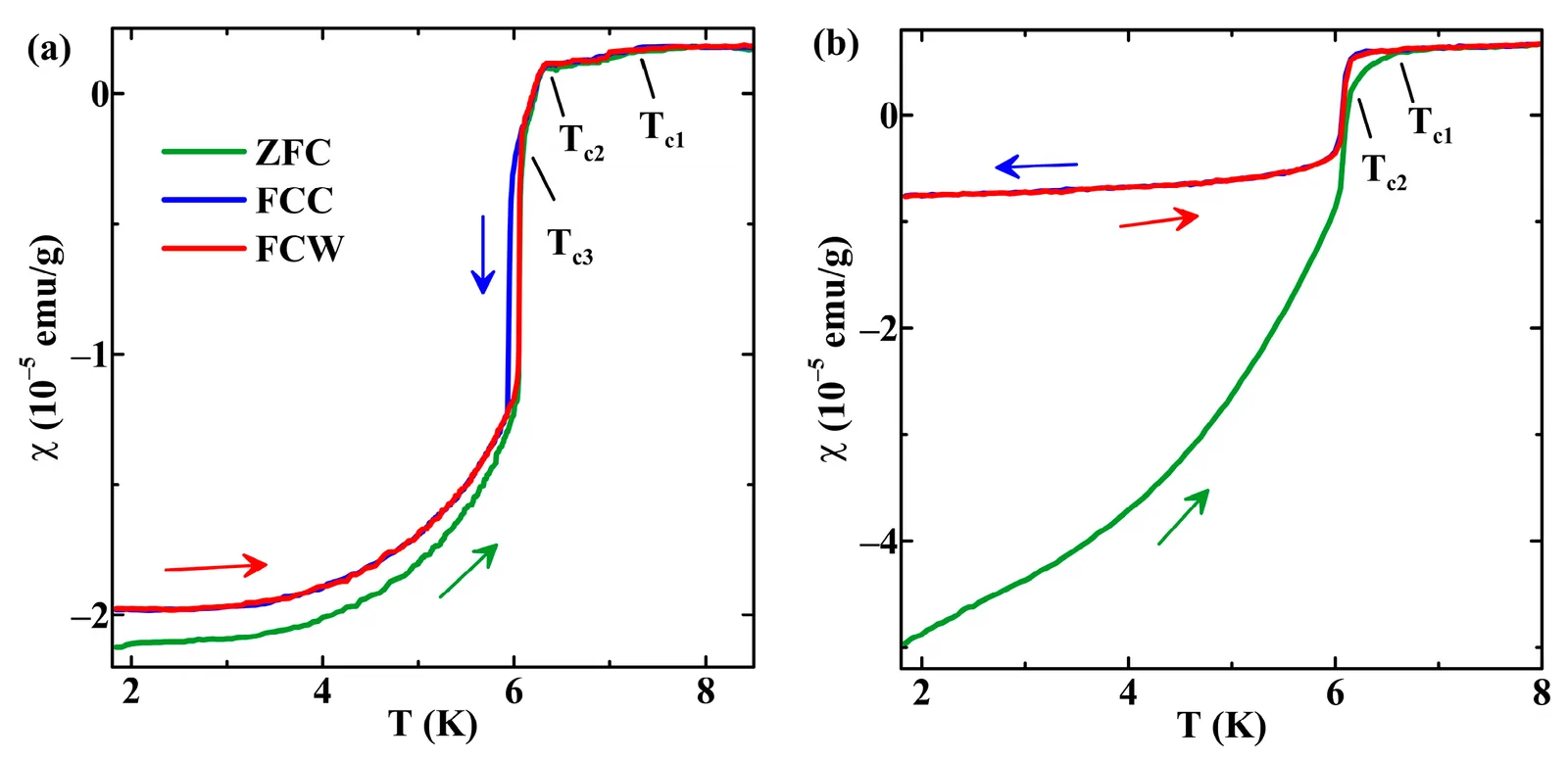
Temperature dependences of susceptibility at 10 Oe for the opal (**a**) and porous glass (**b**) nanocomposites. The directions of increasing and decreasing temperature are shown by arrows.

Variations in susceptibility *χ* with temperature in the 10 Oe field are shown in Figure 5 for the nanocomposites under study. Sharp changes in diamagnetic susceptibility are seen in both samples. The onset of feeble diamagnetism in the 10 Oe field for the opal nanocomposite is at *T_c_*_1_ = 7.15 ± 0.05 K. Slightly more noticeable changes are seen at *T_c_*_2_ = 6.28 ± 0.02 K. The most remarkable diamagnetic screening is observed below *T_c_*_3_ = 6.09 ± 0.02 K. Nevertheless, the relative sample volume screened from the magnetic field, which was estimated from the experimental data, was only near 0.1%. Weak screening indicates that only a small part of the nanoconfined alloy is in the superconducting state. Diamagnetism in the glass nanocomposite at 10 Oe is characterized by two temperatures, *T_c_*_1_ = 6.65 ± 0.02 K and *T_c_*_2_ = 6.15 ± 0.02 K. Minor diamagnetism can also be seen above *T_c_*_1_, likely because of the superconducting fluctuations. Screening at 1.8 K is slightly higher than for the opal nanocomposite and is near 0.25%. Therefore, the superconducting currents envelop only a small part of the total sample volume.

Different steps in the temperature dependences of susceptibility appear due to different superconducting crystalline structures of the Ga-Ag alloy under nanoconfinement. According to the phase diagram of the bulk eutectic Ga-Ag alloy, which has a low silver content [25,26], below the solidus, it consists of intermetallic Ag_3_Ga_2_ segregates [27] with space group *Pmmm* and gallium-rich segregates. The intermetallic Ag_3_Ga_2_ was reported to be nonsuperconducting above 1.6 K [28,29]. The bulk α-Ga has a low superconducting transition temperature of 1.08 K. Thus, the observed superconductivity for both nanocomposites cannot be produced by segregates with the α-Ga crystalline structure, whose formation within pores was demonstrated by X-ray diffraction. The lowest transition temperatures *T_c_*_3_ and *T_c_*_2_ found for the opal and glass nanocomposites, respectively, are quite similar to the superconducting temperature of bulk β-Ga equal to 6.07 K [30]. Thus, we can suggest that the main contribution to superconductivity in both nanocomposites is given by the gallium segregates with the β-Ga structure. The X-ray powder diffraction pattern for the Ga-Ag alloy embedded into the porous glass shows two peaks associated with β-Ga (Figure 4b). The low intensities of the peaks agree with the weak superconductivity in the sample (Figure 5b). Superconductivity in the opal nanocomposite is still weaker than for the glass nanocomposite. Because of the smaller amount of β-Ga, the β-Ga peaks in the X-ray pattern for the alloy within the opal template are probably indistinguishable from the background noise.

Other superconducting transitions should be related to crystalline gallium structures which can occur under nanoconfinement or in nanostructures [9,31,32,33,34,35]. Three specific gallium crystalline structures were found under nanoconfinement, ι, κ, and λ, with superconducting temperatures of 7.1, 6.7, and 6.3 K, respectively [9]. The temperatures *T_c_*_1_ = 7.15 K and *T_c_*_2_ = 6.28 K obtained for the Ga-Ag alloy embedded into the opal template can then be associated with the gallium segregates with the ι-Ga and λ-Ga symmetry, respectively. The temperature *T_c_*_1_ = 6.65 K observed for Ga-Ag within the porous glass is likely related to the gallium segregates with the κ-Ga symmetry. Although X-ray measurements down to 110 K do not show the ι-, κ-, and λ-Ga polymorphs—likely because of their very small volume fractions—their emergence under nanoconfinement is well confirmed by previous low-temperature X-ray studies [9,35]. The results show that different gallium polymorphs form under various nanoconfinements. More studies are needed to understand the relation between the nanoconfined gallium polymorphism and the morphology of the porous templates. Note that a single superconducting transition accompanied by strong shielding was observed for the Ga-Ag alloy embedded into the 7 nm porous glass. The critical temperature was attributed to ι-Ga [24]. In contrast, three superconducting transitions were found for a Ga-Ag/porous glass nanocomposite with pores of 10 nm [23]. The transitions were ascribed to ι-Ga, β-Ga, and a silver–gallium intermetallic with a critical temperature of 2.65 K.

**Figure 6:**
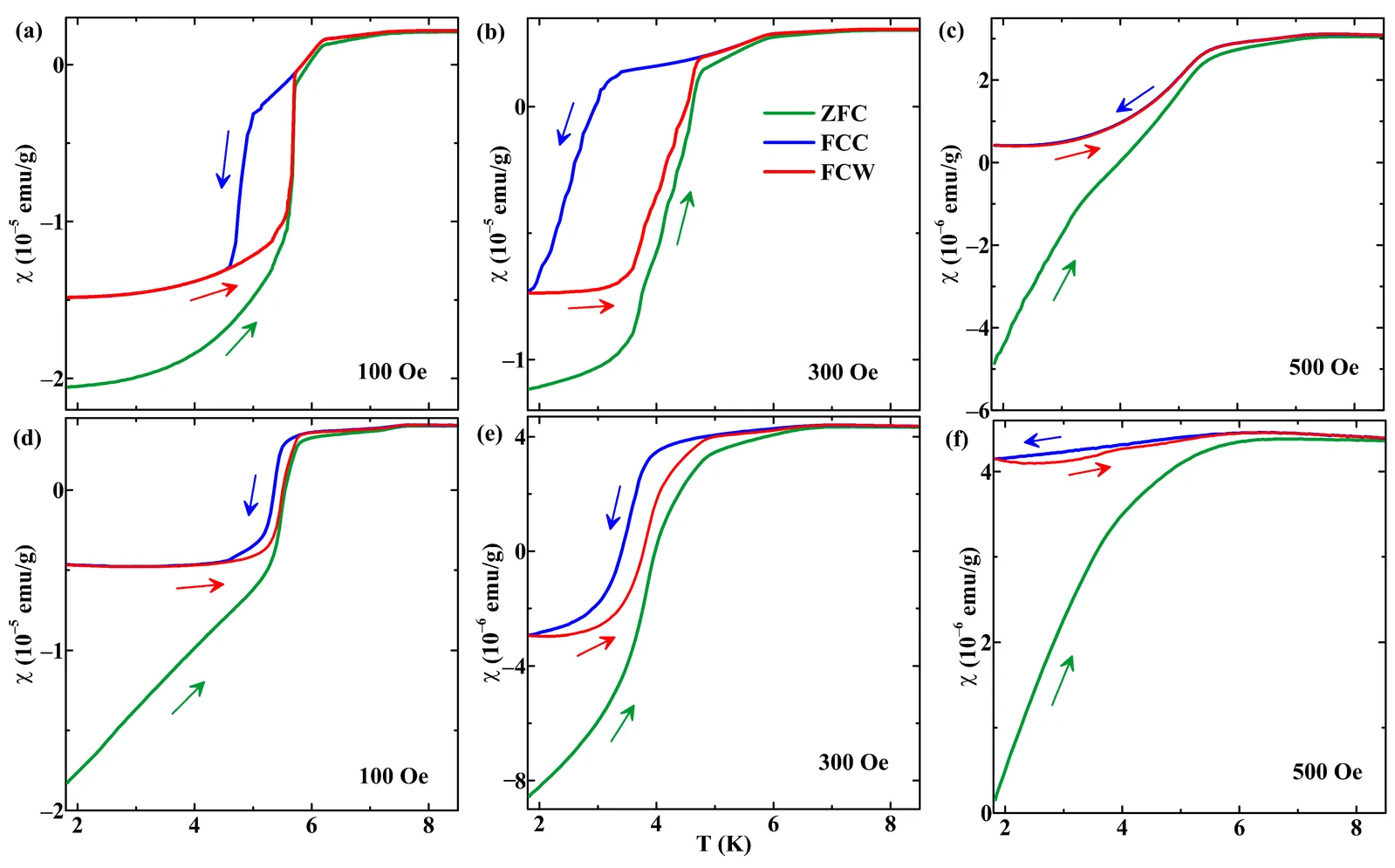
Temperature dependences of susceptibility at 100 Oe (**a**,**d**), 300 Oe (**b**,**e**) and 500 Oe (**c**,**f**) for the opal (**a**–**c**) and glass (**d**–**f**) nanocomposites. The directions of changing temperature are shown by arrows.

**Figure 7:**
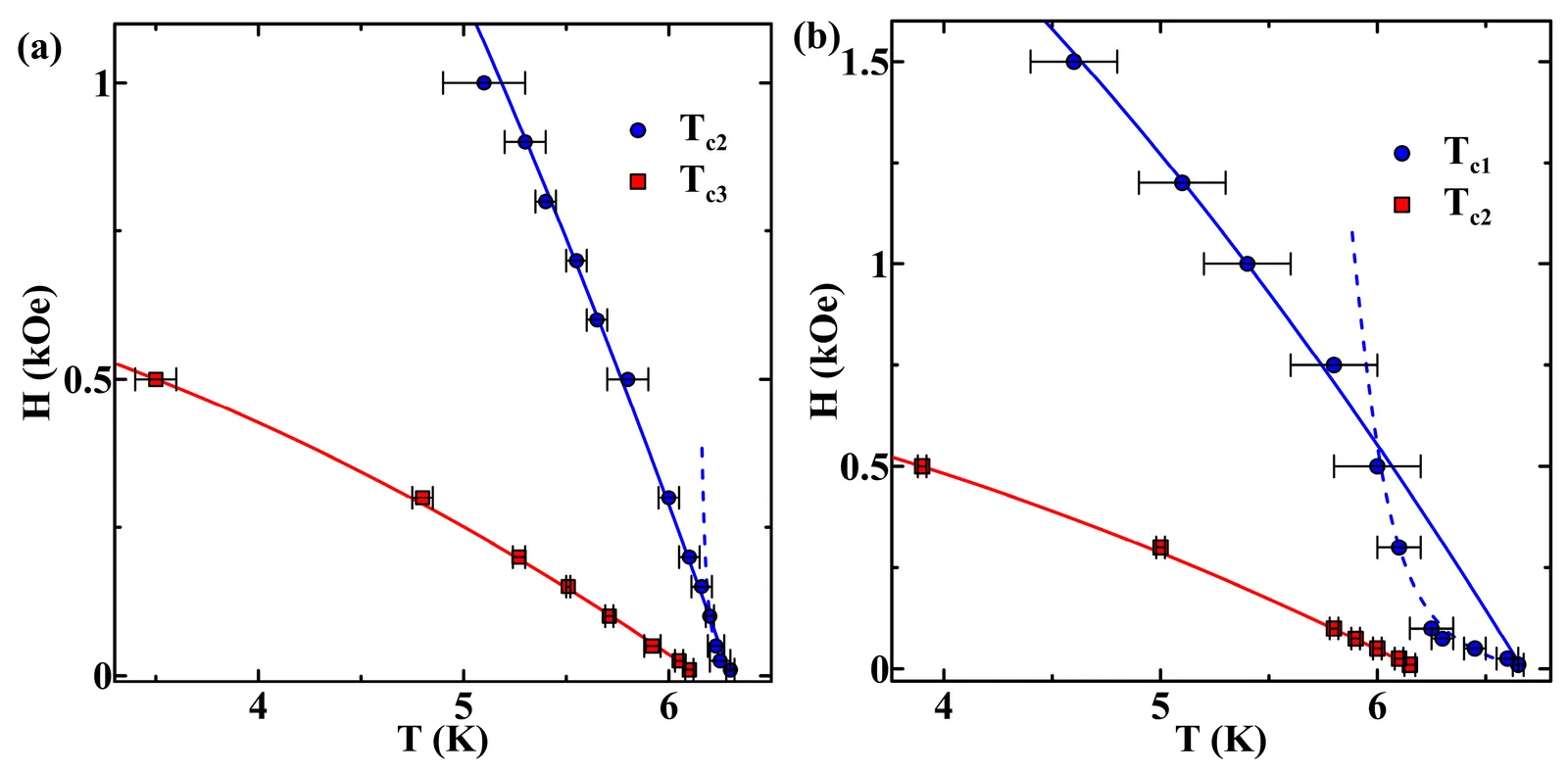
Phase diagrams for the opal (**a**) and porous glass (**b**) nanocomposites. The lines show the theoretical approximations.

The superconducting transitions shift to lower temperatures and become smoother at stronger magnetic fields. The examples for both nanocomposites are shown in Figure 6 for fields of 100, 300, and 500 Oe. The transitions broaden and partly overlap. For the opal nanocomposite, the changes in susceptibility at the first transition flatten out at fields higher than 100 Oe. The critical temperatures observed at different magnetic fields allow us to plot the phase diagrams for the opal and glass nanocomposites with the Ga-Ag alloy for the transitions whose temperatures can be evaluated. They are shown in Figure 7. The critical lines for the first transition in the glass nanocomposite and for the second transition in the opal nanocomposite consist of two segments with positive and negative curvatures.

Upward segments were observed in various bulk type-II superconductors and in some composites with metallic nanoparticles (see [17,18,23,24] and references therein). It was suggested in [17] that the upturn in the temperature dependence of the critical field is related to their morphology, which can be modeled as granular superconductors. The proximity effect was found in [36] to cause the positive curvature of the upper critical line to transform to the negative one with increasing field. Using the relationships obtained in [36], we fitted the segments on the critical lines as shown in Figure 7.

The higher-field segments of the critical lines for the first transition in the glass nanocomposite and the second transition in the opal nanocomposite, as well as the critical lines on the phase diagram for the transitions in β-Ga, show the ordinary negative curvature (Figure 7). A fit using the two-fluid model gives the estimates 3.1 kOe and 740 Oe for the zero-temperature critical fields for the second and third transitions in the Ga-Ag alloy within the opal template, respectively. The estimate for the third transition is close to the 570 Oe value of the critical field for bulk β-Ga [30]. A rather similar result was reported for Ga-Ag dendritic particles embedded into a porous glass with pores of 10 nm [23]. The upper critical field for λ-Ga was not reported.

For the glass nanocomposite under study, the two-fluid model estimates for the first and second transitions are 2.9 kOe and 830 Oe, respectively. The estimate for the β-Ga segregates is also rather similar to the critical field in the bulk β-Ga. The data for κ-Ga were not reported.

**Figure 8:**
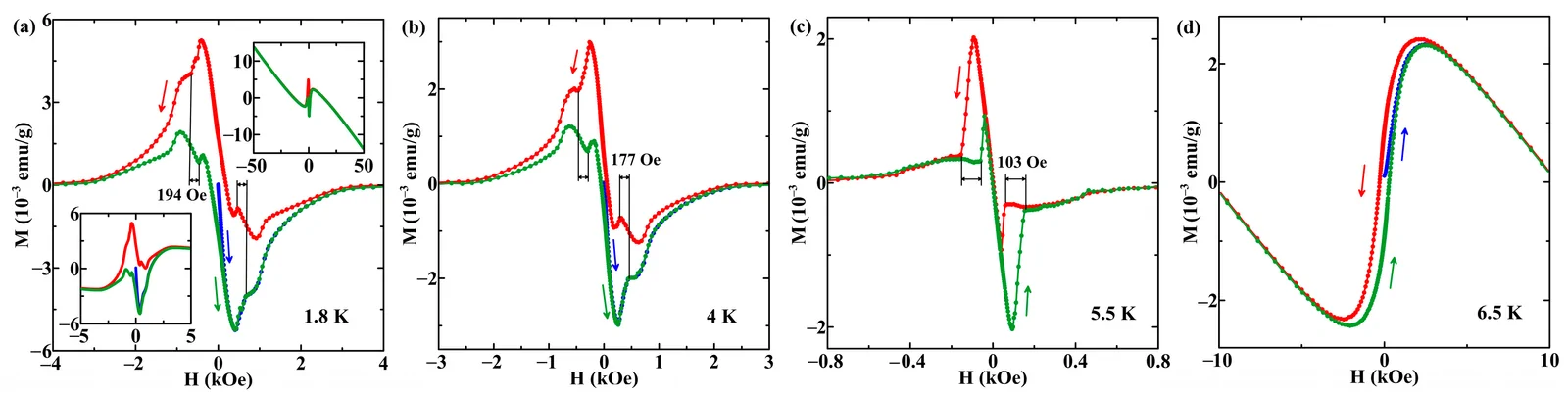
Magnetization isotherms for the opal nanocomposite at temperatures shown in the panels. In panels (**a**–**c**) the ferromagnetic contribution is excluded. The insets to (**a**) show the hysteresis loops on a larger scale. The arrows indicate the direction of the magnetic field sweep.

**Figure 9:**
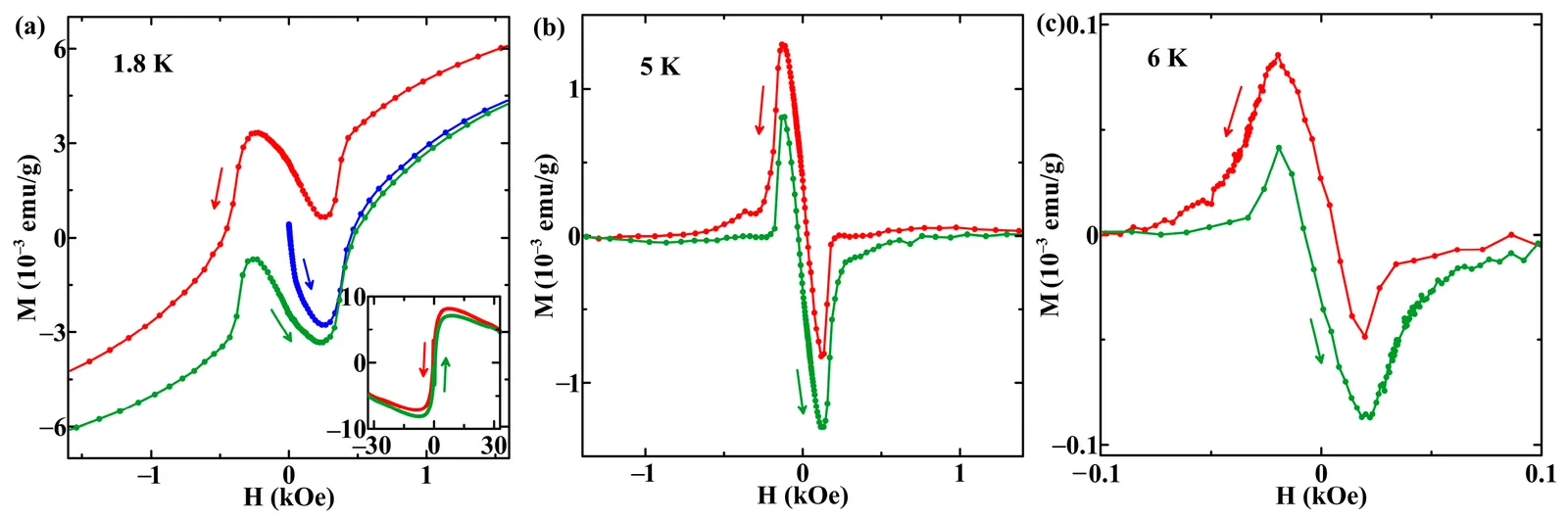
Magnetization isotherms for the porous glass nanocomposite at temperatures shown in the panels. In panels (**b**,**c**) the ferromagnetic contribution is excluded. The inset to (**a**) shows the total hysteresis loop. The arrows indicate the direction of the magnetic field sweep.

Figure 8 and Figure 9 show the magnetization isotherms at several temperatures for the opal and porous glass nanocomposites. The insets to Figure 8a and Figure 9a justify that the magnetization isotherms are the superposition of hysteresis loops caused by superconductivity and ferromagnetism. Ferromagnetism persists above the superconducting transitions up to room temperature. An example of the ferromagnetic loop is presented in Figure 8d. At higher fields the diamagnetic contribution dominates. Ferromagnetism weakly depends on temperature. This allows us to separate the loops induced by superconductivity from the background of the magnetic ordering. Figure 8a–c and Figure 9b,c show the magnetization isotherms resulting from the total hysteresis loops without the ferromagnetic contribution. The critical magnetic fields in the hysteresis loops correlate with those in the phase diagrams.

The magnetization isotherms for the opal nanocomposite in Figure 8 consist of two superconducting hystereses due to λ-Ga and β-Ga. The impact of ι-Ga is too weak to be seen in the hysteresis loops. In agreement with the phase diagram, the central part of the loops is related to the segregates with the β-Ga structure. The magnetization loops for β-Ga are quite reversible—this is clearly seen at 5.5 K. The shapes of the hystereses are similar to those in type-I superconductors, which were analyzed in detail in [37]. Taking into account that the critical fields for the β-Ga segregates are only slightly higher than in bulk β-Ga, we can suggest that the β-gallium network under nanoconfinement is thoroughly interconnected, leading to a small decrease in the correlation length. The β-Ga dendritic particles occupy several neighboring pores in the opal template, in agreement with the narrow peaks in the X-ray patterns, and remain type-I superconductors. The wider constituent loop originates due to λ-Ga. The isotherms are partly reversible with critical fields correlating with the phase diagrams. The loops have a shape typical of type-II superconductors with weak pinning. The high critical fields seen in the phase diagram and hysteresis loops confirm the type-II superconductivity.

The division of the magnetization isotherms into two hysteresis loops for the glass nanocomposite (Figure 9) is not as obvious. Nevertheless, we can assume—using the above-cited data on critical fields—that the central parts of the loops appear owing to the β-Ga segregates. The shape and critical field agree with type-I superconductivity, which survives under nanoconfinement because the dendritic particles extend over adjacent pores. The wider hysteresis constituent corresponds to κ-Ga. The shape of the hysteresis and high critical field correspond to type-II superconductivity.

Figure 8 shows that the virgin, secondary and tertiary branches of the central parts in the magnetization isotherms are shifted relative to each other. This shift indicates the interplay between superconductivity and ferromagnetism. Ferromagnetism was not found in the bulk Ga-Ag alloy [28,29]. However, the emergence of magnetic ordering was reported for low-dimensional elemental metals, including silver nanoparticles and thin films (see [38,39] and references therein). Theoretical models predicted that ferromagnetism in nanosystems appears due to uncompensated spins on the surface. We can suggest that ferromagnetism in the Ga-Ag alloy under nanoconfinement emerges at the interface between the gallium and Ag_3_Ga_2_ intermetallic segregates. The relative shifts of the branches in the magnetization isotherms are less evident for the Ga-Ag alloy in the glass nanocomposite (Figure 9b,c). Note that ferromagnetism was not observed in previous studies of the Ga-Ag alloy under glass nanoconfinement [23,24].

The coexistence of superconductivity and ferromagnetism was recently observed in low-dimensional systems [40,41,42]. The proximity effects yield specific phenomena such as a shift in the Fraunhofer oscillations in the critical current upon increasing and decreasing magnetic field. The shifts between the secondary and tertiary branches in the magnetization isotherms for the In-Ag eutectic alloy within a nanoporous glass were also ascribed to the proximity of superconducting and ferromagnetic regions [20,21]. The impact of the magnetic stray fields and the short-range inverse and electromagnetic proximity effects were discussed in [40,42,43]. The dominant proximity effect influences the sign of the above-mentioned shifts. If the major effect is due to the stray fields, the secondary branch of the magnetization isotherm moves to negative fields because of the positive remanence. In contrast, the tertiary branch moves to positive fields. The opposite sign of the shifts should be observed for the dominance of the other two proximity effects. Figure 8 proves the prevalence of the influence of the remanent magnetization in the ferromagnetic regions. The same behavior was found for the In-Ag alloy embedded into a porous glass template [20,21].

The hysteresis between the FCC and FCW curves (Figure 5a and Figure 6) was very reproducible. The FCW susceptibility approaches the ZFC curve in such a way that the hysteresis loop is formed by the FCC susceptibility on the low-temperature side and by merged FCW and ZFC curves on the high-temperature side. Such hysteresis loops were predicted in [44] using the critical current concept and were occasionally reported for some type-II superconductors [45] and for the nanocomposite with Ga-Ag [24]. However, they were found very rarely.

## 4. Conclusions

We showed that particular nanoconfinement leads to a pronounced increase in the superconducting transition temperatures for the Ga-Ag alloy whose composition was close to the eutectic point of the bulk counterpart. The increased critical temperatures and the emergence of multiple superconducting transitions were attributed to the enhanced polymorphism in the gallium segregates within the opal and glass matrices. The major contribution to superconductivity was provided by the β-Ga polymorph. The β-Ga segregates within nanopores remain type-I superconductors, likely due to the strong interconnection with the alloy in adjacent pores. Both nanocomposites with the Ga-Ag alloy demonstrated ferromagnetic ordering, which coexisted with superconductivity at low temperatures. Ferromagnetism was suggested to be related to uncompensated spins at the interface between the gallium and intermetallic Ag_3_Ga_2_ segregates. The branches in the magnetization isotherms were found to be shifted relative to each other because of the remanent magnetization. A reproducible hysteresis between the FCC and FCW susceptibility curves was observed, which was caused by pinning vortices.

## Data Availability

The raw data supporting the conclusions of this article will be made available by the authors on request.

## References

[1] Li Z., Han X., Guo X., Ma L., Sun J., Wen Y., Guo Y. (2026). Gallium-Based Liquid Metals: From Properties to Applications. Nanomaterials.

[2] Qu C.-C., Liang Y.-T., Wang X.-Q., Gao S., He Z.-Z., Sun X.-Y. (2022). Gallium-Based Liquid Metal Materials for Antimicrobial Applications. Bioengineering.

[3] Bark H., Lee P.S. (2024). Flexible and Printable Composite Ink for Thermal Management of Soft Electronics. Adv. Funct. Mater..

[4] Song H., Kim T., Kang S., Jin H., Lee K., Yoon H.J. (2019). Ga-Based Liquid Metal Micro/Nanoparticles: Recent Advances and Applications. Small.

[5] Akyildiz K., Kim J.-H., So J.-H., Koo H.-J. (2022). Recent progress on micro- and nanoparticles of gallium-based liquid metals: From preparation to applications. J. Ind. Eng. Chem..

[6] Narlikar A.V. (2017). The Oxford Handbook of Small Superconductors.

[7] Wordenweber R., Moshchalkov V., Bending S., Tafuri F. (2017). Superconductors at the Nanoscale: From Basic Research to Applications.

[8] Lee M.K., Tien C., Charnaya E.V., Sheu H.-S., Kumzerov Y.A. (2010). Structural variations in nanosized confined gallium. Phys. Lett. A.

[9] Charnaya E.V., Tien C., Lee M.K., Kumzerov Y.A. (2009). Superconductivity and structure of gallium under nanoconfinement. J. Phys. Condens. Matter.

[10] Tien C., Charnaya E.V., Wang W., Kumzerov Y.A., Michel D. (2006). Possible liquid-liquid transition of gallium in opal. Phys. Rev. B.

[11] Vasilev A.A., Nefedov D.Y., Charnaya E.V., Kumzerov Y.A., Fokin A.V. (2024). Impact of Porous Matrix Morphology on the Phase Diagrams in the GaInSn Alloy Under Nanoconfinement. Appl. Magn. Reson..

[12] Bao W., Zhang J., Rao W., Liu J. (2025). Liquid metal printed superconducting circuits. Phys. Scr..

[13] Yao Z., Sandberg M., Abraham D.W., Bishop D. (2024). Low-loss liquid metal interconnects for superconducting quantum circuits. Appl. Phys. Lett..

[14] Herrmannsdörfer T., Heera V., Ignatchik O., Uhlarz M., Mucklich A., Posselt M., Reuther H., Schmidt B., Heinig K.-H., Skorupa W. (2009). Superconducting state in a gallium-doped germanium layer at low temperatures. Phys. Rev. Lett..

[15] Heera V., Mücklich A., Posselt M., Voelskow M., Wündisch C., Schmidt B., Skrotzki R., Heinig K.-H., Herrmannsdörfer T., Skorupa W. (2010). Heavily Ga-doped germanium layers produced by ion implantation and flash lamp annealing: Structure and electrical activation. J. Appl. Phys..

[16] Ren L., Zhuang J., Casillas G., Feng H., Liu Y., Xu X., Liu Y., Chen J., Du Y., Jiang L. (2016). Nanodroplets for stretchable superconducting circuits. Adv. Funct. Mater..

[17] Lee M.K., Charnaya E.V., Mühlbauer S., Jeng U., Chang L.J., Kumzerov Y.A. (2021). The morphologic correlation between vortex transformation and upper critical field line in Opal-based nanocomposites. Sci. Rep..

[18] Lee M.K., Charnaya E.V., Tien C., Chang L.J., Kumzerov Y.A. (2013). Ac susceptibility studies of a superconducting gallium nanocomposite. J. Appl. Phys..

[19] Uskov A.V., Charnaya E.V., Kuklin A.I., Lee M.-K., Chang L.-J., Kumzerov Y.A., Fokin A.V. (2023). Ga-In alloy segregation within a porous glass as studied by SANS. Nanomaterials.

[20] Likholetova M.V., Charnaya E.V., Shevchenko E.V., Kumzerov Y.A., Fokin A.V. (2024). Coexistence of Superconductivity and Magnetic Ordering in the In–Ag Alloy Under Nanoconfinement. Nanomaterials.

[21] Likholetova M.V., Charnaya E.V., Lozanskaya P.D., Kumzerov Y.A., Fokin A.V. (2025). Superconductivity of the In−Ag alloy embedded into a porous glass. Phys. Solid State.

[22] Bag S., Bhattacharya S., Dinda D., Jyothirmai M.V., Thapa R., Saha S.K. (2018). Induced ferromagnetism and metal-insulator transition due to a charge transfer effect in silver nanoparticle decorated MoS_2_. Phys. Rev. B.

[23] Smetanin D.V., Likholetova M.V., Charnaya E.V., Lee M.K., Chang L.J., Shevchenko E.V., Kumzerov Y.A., Fokin A.V. (2022). Superconductivity and Phase Diagram in the Nanostructured Eutectic Ga-Ag Alloy. Phys. Solid State.

[24] Shevchenko E.V., Charnaya E.V., Lee M.K., Chang L.-J., Likholetova M.V., Lezova I.E., Kumzerov Y.A., Fokin A.V. (2020). Superconductivity in a Ga-Ag nanocomposite with dendritic morphology. Physica C.

[25] Weibke F., Meisel K., Weigels L. (1932). Das Zustandsdiagramm des Systems Silber-Gallium. Z. Anorg. Allg. Chem..

[26] Predel B., Stein D.W. (1972). Bildungsenthalpien binärer Verbindungen des Galliums mit Kupfer, Silber und Gold sowie Analyse der thermodynamischen Eigenschaften von 3/2-Elektronen-Verbindungen. Acta Metall..

[27] Zhang Y., Li J.-B., Liang J.K., Liu Q.L., Xiao Y.G., Zhang Q., Rao G.H., Li C.R. (2006). Thermodynamic assessment of the Ag–Ga system. Calphad.

[28] Alekseevskii N.E. (1966). Superconductivity in the Ag-Ga system. Sov. Phys. JETP.

[29] Hoyt R.F., Mota A.C., Luengo C.A. (1976). Low-temperature specific heat and superconductivity of α-phase Au−Ga alloys. Phys. Rev. B.

[30] Campanini D., Diao Z., Rydh A. (2018). Raising the superconducting T_c_ of gallium: In situ characterization of the transformation of α-Ga into β-Ga. Phys. Rev. B.

[31] Di Cicco A. (1998). Phase Transitions in Confined Gallium Droplets. Phys. Rev. Lett..

[32] He H., Fei G.T., Cui P., Zheng K., Liang L.M., Li Y., Zhang L.D. (2005). Relation between size and phase structure of gallium: Differential scanning calorimeter experiments. Phys. Rev. B.

[33] Liu Z., Bando Y., Mitome M., Zhan J. (2004). Unusual Freezing and Melting of Gallium Encapsulated in Carbon Nanotubes. Phys. Rev. Lett..

[34] Teske D., Drumheller J.E. (1999). Phases of gallium nucleated by small particles. J. Phys. Condens. Matter.

[35] Charnaya E.V., Tien C., Lin K.J., Wur C.-S., Kumzerov Y.A. (1998). Superconductivity of gallium in various confined geometries. Phys. Rev. B.

[36] Theodorakis S., Teanovic Z. (1989). Positive curvature of H_c2_ in layered superconductors. Phys. Rev. B.

[37] Provost J., Paumier E., Fortini A. (1974). Shape Effects on the Magnetization of Superconducting Lead at 4.2K. J. Phys. F Met. Phys..

[38] Sakamoto Y., Oba Y., Maki H., Suda M., Einaga Y., Sato T., Mizumaki M., Kawamura N., Suzuki M. (2011). Ferromagnetism of Pt Nanoparticles Induced by Surface Chemisorption. Phys. Rev. B.

[39] Marenco A.J., Pedersen D.B., Trudel S. (2017). On the origin of the ferromagnetic signature in silver nanoparticles and thin films. J. Mater. Chem. C.

[40] Satariano R., Parlato L., Vettoliere A., Caruso R., Ahmad H.G., Miano A., Palma L.D., Salvoni D., Montemurro D., Granata C. (2021). Inverse Magnetic Hysteresis of the Josephson Supercurrent: Study of the Magnetic Properties of Thin Niobium/Permalloy (Fe_20_Ni_80_) Interfaces. Phys. Rev. B.

[41] Kapran O.M., Golod T., Iovan A., Sidorenko A.S., Golubov A.A., Krasnov V.M. (2021). Crossover between Short- and Long-Range Proximity Effects in Superconductor/Ferromagnet/Superconductor Junctions with Ni-Based Ferromagnets. Phys. Rev. B.

[42] Fermin R., Van Dinter D., Hubert M., Woltjes B., Silaev M., Aarts J., Lahabi K. (2022). Superconducting Triplet Rim Currents in a Spin-Textured Ferromagnetic Disk. Nano Lett..

[43] Buzdin A.I. (2005). Proximity Effects in Superconductor-Ferromagnet Heterostructures. Rev. Mod. Phys..

[44] Clem J.R., Hao Z. (1993). Theory for the hysteretic properties of the low-field dc magnetization in type-II superconductors. Phys. Rev. B.

[45] Sundar S., Chattopadhyay M.K., Chandra L.S.S., Roy S.B. (2015). High field paramagnetic Meissner effect in Mo_100−x_Re_x_ alloy superconductors. Supercond. Sci. Technol..

